# Absolute bioavailability and regional absorption of ticagrelor in healthy volunteers

**DOI:** 10.3109/21556660.2014.946604

**Published:** 2014-08-12

**Authors:** Renli Teng, Juan Maya

**Affiliations:** AstraZeneca LP, Wilmington, DEUSA

**Keywords:** Absolute bioavailability, Regional absorption, P2Y_12_ receptor antagonist, Ticagrelor

## Abstract

**Objective:**

Ticagrelor is a direct-acting, reversibly-binding, oral P2Y_12_ receptor antagonist. It demonstrates predictable, linear pharmacokinetics. Two studies were undertaken to further elucidate the absolute bioavailability of ticagrelor and its regional absorption in the gastrointestinal (GI) tract.

**Design and methods:**

In two open-label, randomized, cross-over studies, 12 volunteers received a single dose of ticagrelor: oral 90 mg and 15 mg IV (Study 1); or 100 mg oral suspension vs 100 mg immediate release (IR) tablet (Study 2). After the initial cross-over period in Study 2, patients received 100 mg suspension delivered to specific sites in the GI tract using an Enterion capsule. In both studies, plasma concentrations of ticagrelor and AR-C124910XX were measured following administration of each formulation.

**Results:**

The mean absolute bioavailability of ticagrelor was 36% (95% confidence interval = 30–42%). Metabolite:parent ratios were higher after oral administration, compared with IV administration (maximum plasma concentration [*C*_max_] = 0.356 and 0.037; area under the plasma concentration-time curves [AUC] = 0.530 and 0.173, respectively). Following oral administration of the 100 mg IR tablet, the AUC and *C*_max_ of ticagrelor were 78% and 58%, respectively, of those following oral administration of the 100 mg suspension. Exposure to ticagrelor decreased the further down the GI tract it was released: mean *C*_max_ for ticagrelor was 91%, 68%, and 13% that for the oral suspension when released in the proximal small bowel, distal small bowel and ascending colon, respectively; mean AUCs were 89%, 73%, and 32%, respectively.

**Conclusion:**

The mean absolute bioavailability of ticagrelor was 36% and the proportion of ticagrelor absorbed decreased the further down the GI tract it was released: the mean AUC for ticagrelor was 89% (proximal small bowel), 73% (distal small bowel), and 32% (ascending colon) that of the mean AUC for the orally administered suspension.

## Introduction

Ticagrelor is a reversibly-binding, oral P2Y_12_ receptor antagonist^1^ that also inhibits equilibrative nucleoside transporter-1 (ENT1)-mediated cellular uptake of adenosine^2^. Ticagrelor is approved for the prevention of atherothrombotic events in patients with acute coronary syndromes (ACS). The phase III PLATelet inhibition and patient Outcomes (PLATO) study^3^ showed that ticagrelor (180 mg loading dose followed by 90 mg twice daily) significantly reduced the rate of myocardial infarction, stroke, or death from vascular causes, compared with clopidogrel (both given with aspirin) without increasing the rate of overall major bleeding in patients with ACS^3^.

In early clinical pharmacology studies, ticagrelor demonstrated predictable, linear pharmacokinetics with single oral doses up to 400 mg^4^ and multiple oral doses up to 300 mg twice daily^5^. Ticagrelor was rapidly absorbed, with peak plasma concentrations achieved 1.3–2.0 h after single doses and 1.5–3.0 h after multiple doses^4,5^. Metabolism of ticagrelor is not required for inhibition of platelet aggregation^1^. However, ticagrelor is extensively metabolized^6^ and the active metabolite (AR-C124910XX) formed via cytochrome P450 3A4/5^7^ is present in blood at approximately one third the concentration of the parent compound^6,8^. AR-C124910XX has similar potency to the parent compound with respect to inhibition of the P2Y_12_ receptor (AstraZeneca data on file). In six volunteers receiving a single oral dose of [^14^C]ticagrelor 200 mg as a suspension, 26.5% of total radioactivity was recovered in the urine, with <0.05% of the dose excreted as unchanged ticagrelor or AR-C124910XX, indicating that renal clearance is of minor importance^6^. The majority of radioactivity was recovered in the feces (57.8%), suggesting that ticagrelor has low absorption, or that ticagrelor and its metabolites may have passed directly into the bile from the liver or are excreted into the intestine from the systemic circulation via intestinal P-glycoprotein (P-gP)^6^. Ticagrelor is a substrate and inhibitor of intestinal P-gP (AstraZeneca, data on file)^9^.

Ticagrelor requires twice-daily administration. In order to advance the development of a modified release formulation for a potentially once-daily administration regimen, two studies were undertaken to provide further details regarding the pharmacokinetic profile of ticagrelor in healthy volunteers. Study 1 aimed to determine the absolute bioavailability of oral ticagrelor. Study 2 aimed to determine the regional absorption characteristics of ticagrelor from the proximal small bowel, distal small bowel and ascending colon, and to compare the pharmacokinetic parameters of ticagrelor following oral administration of immediate release (IR) tablet and suspension.

## Methods

### Study populations

In Study 1 (D5130C00038) key inclusion criteria were: healthy volunteers aged 18–45 years, body mass index (BMI) 20–30 kg/m^2^. In Study 2 (D5130C05238) study key inclusion criteria were: healthy volunteers aged 18–65 years, BMI 18–30 kg/m^2^. In both studies female volunteers were required to be post-menopausal or surgically sterile. Key exclusion criteria were a history/current condition known to interfere with drug absorption, distribution, metabolism or excretion; and history of hematological abnormalities or bleeding disorders.

The final study protocols were approved by the New England Institutional Review Board (Study 1) and the Quorn Research Review Committee (Study 2). Both studies were conducted within the ethical principles established in the Declaration of Helsinki, and in accordance with International Conference on Harmonization/Good Clinical Practice guidelines. Applicable regulatory requirements and the AstraZeneca policy on bioethics were followed and written informed consent was provided by all volunteers before enrolment.

### Study designs and treatment

#### Study 1

This study was an open-label, randomized, two-period cross-over study conducted in a single center. Eligibility for study participation was assessed up to 28 days prior to study start. Following a 10-h overnight fast, volunteers were randomized to receive a single oral dose of ticagrelor 90 mg (tablet) and a single IV infusion of ticagrelor 15 mg (150 mL of ticagrelor solution for infusion: 0.1 mg/mL, given at 300 mL/h over 30 min); volunteers fasted for a further 4 h post-dose. Volunteers were discharged from the clinical pharmacology unit (CPU) on day 4 of each treatment and there was a minimum washout period of 7 days between the two treatments. Follow-up was performed 3–5 days after final discharge.

Oral ticagrelor 90 mg was selected as it is the formulation of the approved clinical dose (90 mg twice daily). The 15 mg IV dose was selected based on estimates predicting maximum plasma concentration (*C*_max_) and AUC from a study of absolute bioavailability in marmosets (AstraZeneca, data on file).

#### Study 2

This study was an open-label, randomized, cross-over study conducted in a single center. Eligibility for study participation was assessed up to 21 days prior to study start. Volunteers were randomized to receive a single dose of ticagrelor 100 mg as an oral suspension or a single dose of ticagrelor 100 mg as an immediate release (IR) tablet administered in a fasted state on the morning of Visit 2 and then the alternative treatment at Visit 3. At Visits 4, 5 and 6, ticagrelor 100 mg (suspension) was administered using Enterion capsules (Phaeton Research, Nottingham, UK) configured to release the dose at one of the following gastrointestinal (GI) tract sites during each visit (patients were re-randomized at Visit 4): proximal small bowel, distal small bowel and ascending colon. The Enterion capsule is a remote-controlled drug delivery device containing a gamma-emitting radionuclide tracer port^10^. Each Enterion capsule was administered with 200 mL ^99m^Technetium diethylenetriaminepentaacetic acid to provide an outline of the GI tract. Transit of the capsules through the GI tract was monitored by scintigraphy and, when the capsule reached the target site, it was triggered by a radiofrequency signal to release the ticagrelor suspension. Volunteers received each dose at the CPU, were discharged 24 h after capsule activation and returned 12 h later for assessment. There was a minimum washout period of 96 h between Visits 4–6 and follow-up was performed 7–10 days after final discharge.

Ticagrelor 100 mg was selected based on the size of the drug chamber of the Enterion capsule, and was predicted to provide evaluable pharmacokinetic data beyond 36 h post-dose.

### Pharmacokinetic blood sampling and analyses

In Study 1, venous blood samples (4 mL) were taken pre-dose (0), 0.25, 0.5, 1, 1.5, 2, 2.5, 3, 4, 5, 6, 8, 10, 12, 16, 20, 24, 36, 48 and 72 h post-dosing in each study period.

In Study 2, venous blood samples (4.5 mL) were taken pre-dose (0), 0.5, 1, 1.5, 2, 3, 4, 6, 8, 12, 18, 24 and 36 h after receiving ticagrelor oral suspension or IR tablet (Visits 2 and 3), or following Enterion capsule activation (Visits 4–6; capsule activation equals time zero). All samples were collected into lithium heparin tubes and plasma was separated by centrifugation within 30 min of collection (10 min, 4 °C, 1500 g) and stored frozen (−20 °C) until analyzed.

Ticagrelor and AR-C124910XX plasma concentrations were quantified as described previously^11^. Lower limits of quantification were 5 ng/mL and 2.5 ng/mL for ticagrelor and AR-C124910XX, respectively^11^.

### Assessment of capsule gastrointestinal transit times and regional release of ticagrelor (Study 2)

Anterior scintigraphic images were taken using a gamma camera (General Electric Maxicamera, USA) with a 40 cm field of view and fitted with a medium energy parallel hole collimator. Images were recorded at the following intervals using a Park Medical Micas X computer system: every 10 min until Enterion capsule activation and for 4 h post-activation; every 20 min until 8 h post-activation; every 60 min until 12 h post-activation; and at 18, 24 and 36 h post-activation. Scintigraphic images were visually assessed to determine gastric emptying and target site arrival time. Volunteers provided daily fecal samples for recovery of the Enterion capsule to confirm ticagrelor release.

### Safety and tolerability assessments

The safety and tolerability of ticagrelor were evaluated by assessing the incidence and severity of adverse events (AEs) throughout both studies. In addition, ECGs were performed, and vital signs (blood pressure, heart rate) and clinical laboratory parameters (chemistry, hematology, urinalysis) were assessed throughout both studies.

### Data analyses

Pharmacokinetic parameters for ticagrelor and AR-C124910XX were estimated using non-compartmental methods. The following parameters were estimated in Study 1. *C*_max_ was the highest recorded plasma concentration. *t*_max_ was the time to reach *C*_max_. Terminal elimination half-life (*t*_½_) was calculated as 0.693/*λ_z_*, where *λ_z_*, the terminal-phase elimination rate constant, was calculated by log-linear regression of the terminal portion of the concentration–time profile. AUC was calculated using the linear trapezoid method up to the last measurable concentration (AUC_0–_*_t_*) and thereafter by extrapolation of the terminal elimination phase to infinity. Mean residence time (MRT) was the ratio of area under the first moment–time curve (AUMC) to AUC, after correction for non-instantaneous infusion following IV administration. Total body clearance (CL) after oral and IV dosing of ticagrelor was estimated using the ratio of ticagrelor dose and AUC. The volume of distribution at steady state (*V*_ss_ = CL × AUMC/AUC) was also determined.

Dose-normalized AUC values for oral and IV ticagrelor were logarithmically transformed and were analyzed separately using a mixed effect model; formulation, sequence, and period were fixed effects, and volunteer within sequence was a random effect. Absolute bioavailability (primary endpoint) was calculated as [AUC (oral)/dose (oral)]/[AUC (IV)/dose (IV)]; the primary comparison was oral ticagrelor 90 mg vs IV ticagrelor 15 mg.

In Study 2 the following pharmacokinetic parameters were determined for ticagrelor and AR-C124910XX after administration of the oral suspension, IR tablet and Enterion capsules: *C*_max_, *t*_max_, AUC, AUC_0–_*_t_*, and *t*_½_. No formal statistical tests were performed.

The primary variable was the plasma concentration of ticagrelor and AR-C124910XX following the release of ticagrelor from the Enterion capsule in the proximal and distal small bowel and the ascending colon.

## Results

### Demographic and baseline characteristics

A total of 12 volunteers were enrolled and randomized to receive study drugs in each study.

In Study 1, all participants were male, mean age was 32 years (range = 22–45) and mean BMI was 25.0 (range = 21.0–29.2). The majority were Black (*n* = 9; 75%). All volunteers received IV ticagrelor and one volunteer was enrolled incorrectly and did not receive oral ticagrelor. Eleven volunteers completed the study.

In Study 2, one randomized volunteer was withdrawn due to an AE prior to receiving study drug. Therefore, 11 volunteers received study drugs and all completed the study. All participants were male, the majority were Caucasian (*n* = 10, 91%), mean age was 35 years (range = 18–52), and mean BMI was 25.9 (range = 20.3–29.7).

### Ticagrelor pharmacokinetic parameters (Study 1)

The pharmacokinetic parameters of ticagrelor and AR-C124910XX after oral and IV administration of ticagrelor are shown in Table 1.

**Table 1.** Ticagrelor and AR-C124910XX pharmacokinetic parameters after IV and oral administration.

Parameter	IV ticagrelor (15 mg)	Oral ticagrelor (90 mg)
Ticagrelor
*C*_max_ (ng/mL)	449 (28.9)	403 (38.7)
*t*_max_ (h)	0.48 (0.47–0.49)	1.49 (0.99–4.99)
AUC_0–_*_t_* (ng·h/mL)	1043 (21.2)	2209 (35.8)
AUC (ng·h/mL)	1058 (20.8)	2233 (35.5)
*t*_½_ (h)	6.8 (18.0)	8.1 (16.5)
CL (L/h)	14.2 (20.8)	N/A
*V*_ss_ (L)	87.5 (20.0)	N/A
AR-C124910XX
*C*_max_ (ng/mL)	17 (26.9)	144 (27.4)
*t*_max_ (h)	1.74 (1.24–2.00)	2.00 (1.49–4.99)
AUC_0–_*_t_* (ng·h/mL)	145 (28.0)	1127 (28.5)
AUC (ng·h/mL)	183 (26.3)	1182 (26.7)
*t*_½_ (h)	8.3 (31.7)	8.1 (11.6)
Metabolite: parent *C*_max_ ratio	0.037 (20.2)	0.356 (23.0)
Metabolite: parent AUC ratio	0.173 (22.4)	0.530 (21.1)

Data are geometric mean (coefficient of variation, %) based on log transformed data, except *t*_max_: median (range). AUC, area under the plasma concentration time curve; AUC_0–_*_t_*, AUC from time 0 to last measurable concentration; CL, total body clearance after IV administration; *C*_max_, maximum plasma concentration; N/A, not applicable; *t*_½_, terminal elimination half-life; *t*_max_, time to reach *C*_max_; *V*_ss_, volume of distribution at steady state.

**Figure 1.**
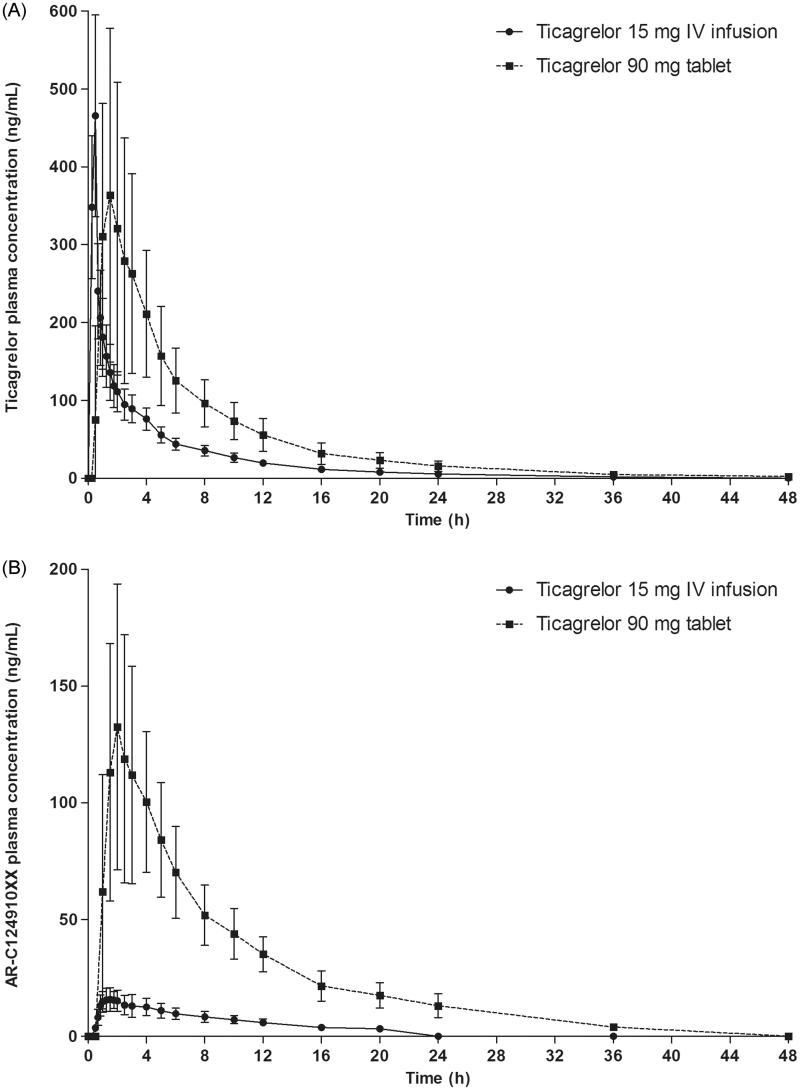
(A) Ticagrelor and (B) AR-C124910XX plasma concentrations following a single oral dose of ticagrelor 90 mg and a 30 min IV infusion of ticagrelor 15 mg.

Ticagrelor was rapidly absorbed after oral administration (median *t*_max_ = 1.49 h) and the median *t*_max_ for ticagrelor following IV administration (0.48 h) coincided with the end of the infusion (Figure 1a; Table 1). The *t*_1/2_ for ticagrelor was similar after IV and oral administration (6.8 vs 8.1 h), and the MRT was also similar between administration routes (6.2 and 8.6 h, respectively). *C*_max_ was similar after oral and IV administration (403 vs 449 ng/mL), while AUC was higher after oral administration (2233 vs 1058 ng h/mL).

The *t*_1/2_ for AR-C124910XX was similar after oral and IV administration of ticagrelor (8.1 h and 8.3 h, respectively). Exposure to AR-C124910XX was higher after oral compared with IV administration of ticagrelor, as were the corresponding metabolite:parent ratios (*C*_max_ = 0.356 and 0.037; AUC = 0.530 and 0.173, respectively).

The mean absolute bioavailability of ticagrelor was 36% (95% confidence interval [CI] = 30–42%), with bioavailability in individuals ranging from 25.4–64.0%.

### Gastrointestinal transit and regional release of ticagrelor (Study 2)

The Enterion capsules exhibited a typical gastric emptying time profile, leaving the stomach within 3 h in the majority of volunteers, and within 5 h in three of four (for proximal small bowel and distal small bowel release) and one of two (for ascending colon release) remaining volunteers. Mean time (standard deviation [SD]) to capsule activation was 2.8 (1.2), 3.9 (1.4), and 6.7 (1.3) h in the proximal small bowel, distal small bowel, and ascending colon, respectively. Capsule activation was successful in the proximal and distal small bowel in all volunteers and in the ascending colon in nine of 11 volunteers (in the remaining two volunteers, retrospective analysis demonstrated that activation occurred in the ileo-cecal junction in one volunteer and the stomach in the other).

### Ticagrelor pharmacokinetic parameters (Study 2)

**Figure 2.**
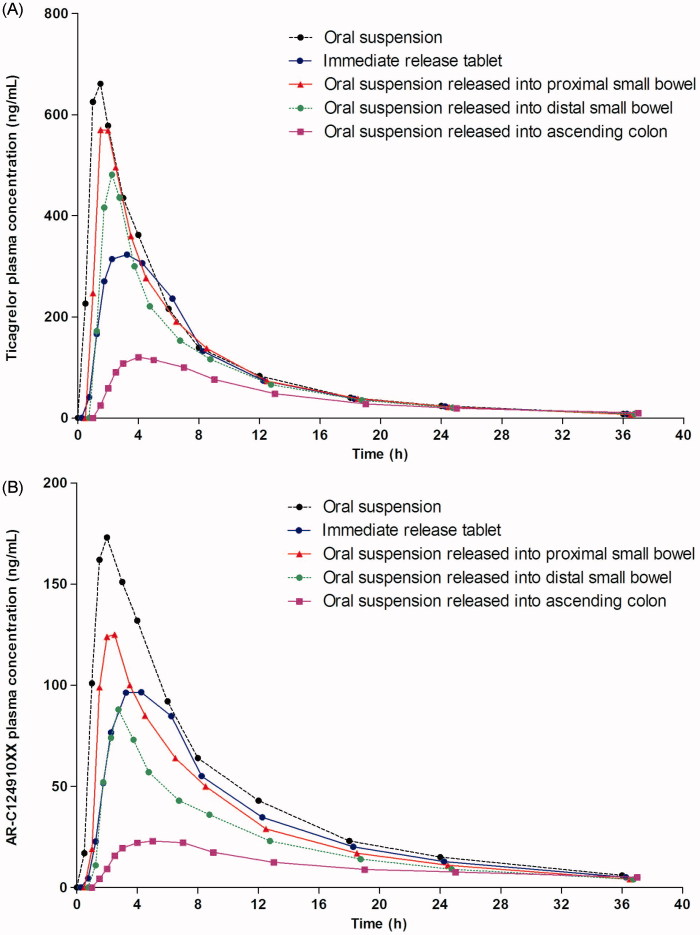
(A) Ticagrelor and (B) ARC124910XX plasma concentrations following a single dose of ticagrelor 100 mg as an oral suspension, immediate release tablet, and a suspension released into the proximal small bowel, distal small bowel, and ascending colon.

Compared with administration of the IR tablet, administration of ticagrelor 100 mg suspension resulted in a higher *C*_max_, AUC and AUC_0–_*_t_* and a shorter *t*_max_, for both ticagrelor and AR-C124910XX (Figure 2; Table 2). The AUC and *C*_max_ of ticagrelor after administration of the IR tablet were 78% and 58%, respectively, of those for the suspension. The *t*_1/2_ for ticagrelor and AR-C124910XX were similar after administration of ticagrelor as an IR tablet and suspension (Table 2).

**Table 2.** Ticagrelor and AR-C124910XX pharmacokinetic parameters following dosing with ticagrelor 100 mg as various oral formulations.

	Ticagrelor 100 mg				
			Enterion tablet released in:		
Parameter	Suspension	IR Tablet	Proximal small bowel	Distal small bowel	Ascending colon
Ticagrelor					
*C*_max_ (ng/mL)	667 (33.8)	384 (20.2)	608 (39.1)	456 (44.5)	85 (90.3)
*t*_max_ (h)	1.50 (1.00–2.00)	3.00 (1.50–6.00)	1.50 (1.00–6.00)	1.50 (1.00–2.02)	4.00 (2.00–8.00)
AUC_0–_*_t_* (ng · h/mL)	3781 (29.0)	2921 (21.1)	3349 (29.4)	2735 (35.7)	1119 (80.5)
AUC (ng · h/mL)	3856 (29.2)	3016 (21.1)	3426 (29.5)	2832 (35.1)	1235 (79.3)
*t*_½_ (h)	7.19 (9.1)	8.08 (22.8)	7.31 (10.3)	7.56 (22.6)	9.68 (32.5)
AR-C124910XX					
*C*_max_ (ng/mL)	174 (27.0)	109 (20.1)	125 (37.4)	77 (57.2)	12 (139.0)
*t*_max_ (h)	2.00 (1.50–4.00)	3.00 (2.00–6.00)	2.00 (1.00–4.00)	2.00 (1.00–3.00)	6.00 (2.00–12.00)
AUC_0–_*_t_* (ng · h/mL)	1480 (21.7)	1086 (22.4)	1050 (27.4)	705 (46.4)	157 (116.6)
AUC (ng · h/mL)	1550 (22.2)	1149 (22.0)	1107 (26.6)	757 (44.9)	435 (78.9)*^a^*
*t*_½_ (h)	8.08 (19.9)	8.57 (14.6)	8.36 (12.0)	8.99 (17.6)	16.04 (42.7)*^a^*

Data are geometric means (coefficient of variation, %), except *t*_max_: median (range). *^a^* Data are for eight volunteers, all other data are for 11 volunteers. AUC, area under the plasma concentration time curve; AUC_0–_*_t_*, AUC from time 0 to last measurable concentration; *C*_max_, maximum plasma concentration; *t*_max_, time to reach *C*_max_; *t*_½_, terminal elimination half-life.

Release of ticagrelor 100 mg suspension in different parts of the GI tract demonstrated that exposure to ticagrelor decreased the further down the GI tract it was released. Following release into the proximal small bowel, distal small bowel, and ascending colon, the mean *C*_max_ for ticagrelor was 91%, 68%, and 13%, respectively, that of the mean *C*_max_ for the orally administered suspension; the mean AUC for ticagrelor was 89%, 73%, and 32%, respectively, of the mean AUC for the orally administered suspension. When ticagrelor was released into the ascending colon, absorption (*t*_max_) of ticagrelor was delayed (mean 3.8 h) and the *t*_½_ (9.7 h) was longer, compared with the administration of the orally administered suspension or release in the proximal and distal small bowel (mean *t*_max _= 1.42–1.51 h; *t*_½_ = 7.19–7.56). Similar trends were observed for AR-C124910XX (Figure 2b; Table 2).

### Safety and tolerability

Ticagrelor was generally well tolerated in both studies. All AEs were of mild intensity and there were no serious AEs, discontinuations due to AEs, or deaths during either study.

In Study 1, five AEs were reported in three volunteers and all but one had resolved by the end of the study. Two volunteers had AEs after receiving oral ticagrelor: rhinorrhea 1 day post-dose and eye pruritis 4 days post-dose, which had not resolved by study end (*n* = 1); pharyngolaryngeal pain 11 days post-dose (*n* = 1). An additional volunteer had headache 1 day after oral dosing and injection-site erythema 2 days after IV dosing, which was considered drug related.

In Study 2, six AEs were reported in five volunteers who received ticagrelor. Two volunteers reported AEs following administration of the ticagrelor IR tablet (headache, paresthesia). Three volunteers reported four AEs following administration of ticagrelor suspension in the Enterion capsule: epistaxis, which was considered drug related (proximal small bowel release); fatigue and acne (distal small bowel release); bleeding post-vessel puncture, which was considered drug related (ascending colon release). No AEs were reported following administration of the ticagrelor oral suspension.

There were no clinically meaningful changes observed in clinical laboratory parameters, ECG, vital signs or physical examinations during either study.

## Discussion

Two studies were undertaken to provide further details regarding the pharmacokinetic profile of ticagrelor in healthy volunteers. The first study found that the mean absolute bioavailability of ticagrelor was 36% (95% CI = 30–42). This result is consistent with findings from a study using a single oral dose of [^14^C]ticagrelor 200 mg in six volunteers in whom the majority of radioactivity was recovered in the feces (57.8%), suggesting that ticagrelor has low bioavailability^6^, passed directly into the bile from the liver or was excreted into the intestine via intestinal P-gP. However, neither our current study nor previous studies have determined the proportion of an administered ticagrelor dose that enters the GI tract via intestinal P-gP transport or via biliary excretion.

The second study was undertaken to compare the absorption profile of ticagrelor following oral administration as an IR tablet vs suspension and to examine the regional absorption of ticagrelor in the GI tract. Compared with administration of the IR tablet, administration of 100 mg ticagrelor as a suspension resulted in a greater exposure to both ticagrelor and AR-C124910XX. Enterion capsules containing 100 mg ticagrelor (suspension) were used to assess regional absorption and generally demonstrated typical GI transit times, given the large inter-subject and intra-subject variability in GI transit with single-unit dosage forms^12–15^. Exposure to ticagrelor and AR-C124910XX decreased the further down the GI tract that ticagrelor was released from the capsule. The largest proportion of ticagrelor was absorbed when it was released in the proximal small bowel, with a pharmacokinetic profile similar to oral administration of the suspension. In addition, the *t*_max_ was 1.5 h after oral administration of ticagrelor suspension, as well as after release of the suspension within the proximal or distal small bowel (via Enterion capsule), suggesting that the rate of absorption of ticagrelor after oral administration is similar throughout the small bowel. Given the low pH-independent solubility and low permeability of ticagrelor^16^, these results are not unexpected. Both permeability and solubility are important factors in colonic drug absorption in humans, and drugs with low permeability generally exhibit reduced absorption after colonic vs oral and proximal small bowel administration^17^. For example, reduced absorption of low permeability and low solubility drugs from the colon may be due to the reduced surface area (i.e., lack of villi) and a greater tight junctional area in the colon compared with the small bowel^18^. Equally, we cannot rule out that ticagrelor was well absorbed from the colon but then metabolized before it reached the systemic circulation; although cytochrome P450 levels tend to be lower in the colon than intestine and PgP-mediated efflux appears to have little impact on colonic absorption in humans^17^.

The pharmacokinetics of oral ticagrelor in Study 1 were consistent with previous reports^4,5^, where rapid absorption of ticagrelor (*t*_max_ = 1.5 h) was consistent with the rapid onset of platelet inhibition^19^. This was the first study to determine the pharmacokinetics of ticagrelor following IV administration. The difference in AUC between a single oral and IV dose of ticagrelor reflects the difference in the dose administered and the impact of first-pass metabolism on the bioavailability of oral ticagrelor. The AR-C124910XX:ticagrelor ratios for exposure were much higher after oral than IV administration, suggesting that the majority of AR-C124910XX formation after oral administration of ticagrelor occurs during absorption and during first pass metabolism.

Ticagrelor was well tolerated, irrespective of the formulation given (oral suspension, IR tablet, IV, suspension within an Enterion capsule). All AEs were mild and all but one resolved by the end of the study (eye pruritus) across both studies.

Our studies were not without limitations. The study populations differed in their racial mix, with Black volunteers comprising 75% of the population in Study 1 and Caucasians comprising 91% of those in Study 2. However, ticagrelor pharmacokinetics are not markedly affected by race^20^, and, in both studies, the volunteers acted as their own controls, which would minimize the impact of racial differences on variation. Our studies included a relatively small number of volunteers, which, when combined with the low absorption of ticagrelor, may have contributed to observed variability in the pharmacokinetic parameters of ticagrelor. Overall, our findings have limited clinical applicability for physicians and pharmacists. However, they provide important insights into the absorption profile of ticagrelor, which will inform the development of a modified release formulation.

In conclusion, the mean absolute oral bioavailability of ticagrelor was 36% (95% CI = 30–42%). Ticagrelor was absorbed from the proximal small bowel, distal small bowel, and ascending colon, and the proportion of ticagrelor absorbed decreased the further down the GI tract it was released: the mean AUC for ticagrelor was 89% (proximal small bowel), 73% (distal small bowel), and 32% (ascending colon) that of the mean AUC for the orally administered suspension. Ticagrelor was well tolerated when administered by IV infusion, and orally as a tablet, suspension, and a suspension released at different regions of the GI tract.
